# Supplementary material for: Exercise Inhibits Doxorubicin-Induced Damage to Cardiac Vessels and Activation of Hippo/YAP-Mediated Apoptosis
Source: Cancers (Basel). 2021 Jun 1;13(11):2740. doi: 10.3390/cancers13112740 (PMC8198139; doi:10.3390/cancers13112740)
Supplement: Supplementary file 1 [file cancers-13-02740-s001.zip › Supplemental Figures.pdf]

Supplemental Figure 1

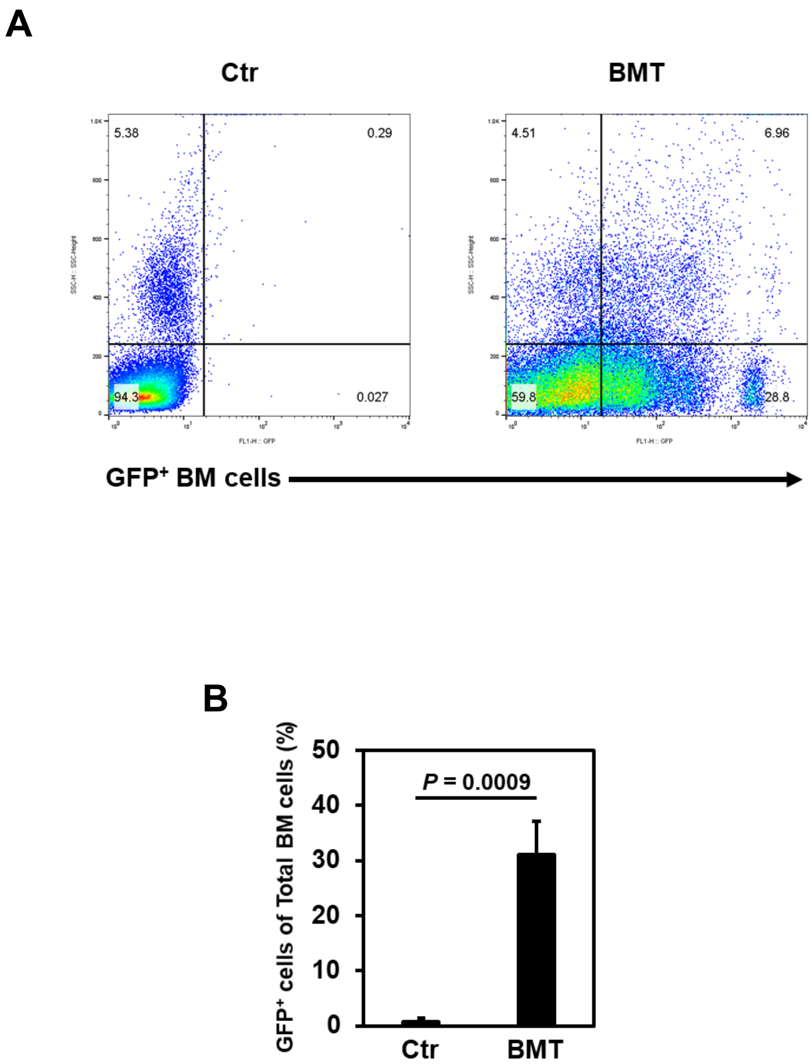

Supplemental Figure 1. Bone marrow transplant (BMT) efficiency.

Representative FACS plots (**A**) and relative quantification (**B**) of GFP positive cells in total BM cells isolated from Ctr and BMT group mice. N=3 mice. Values represent mean±SEM. *P* values are indicated by the Student's *t* test. Ctr, control; BMT, bone marrow transplant.

Supplemental Figure 2

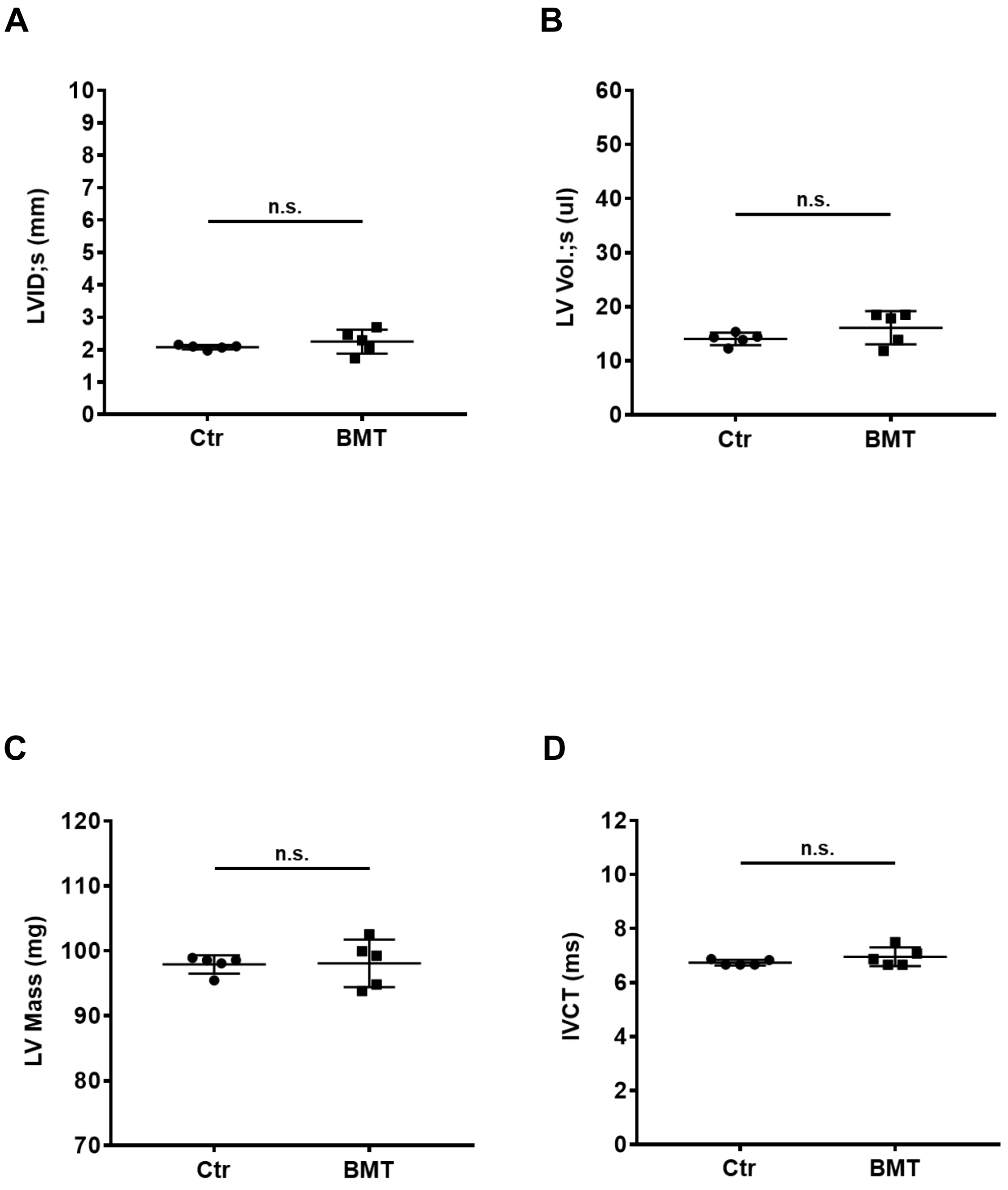

Supplemental Figure 2 (continued)

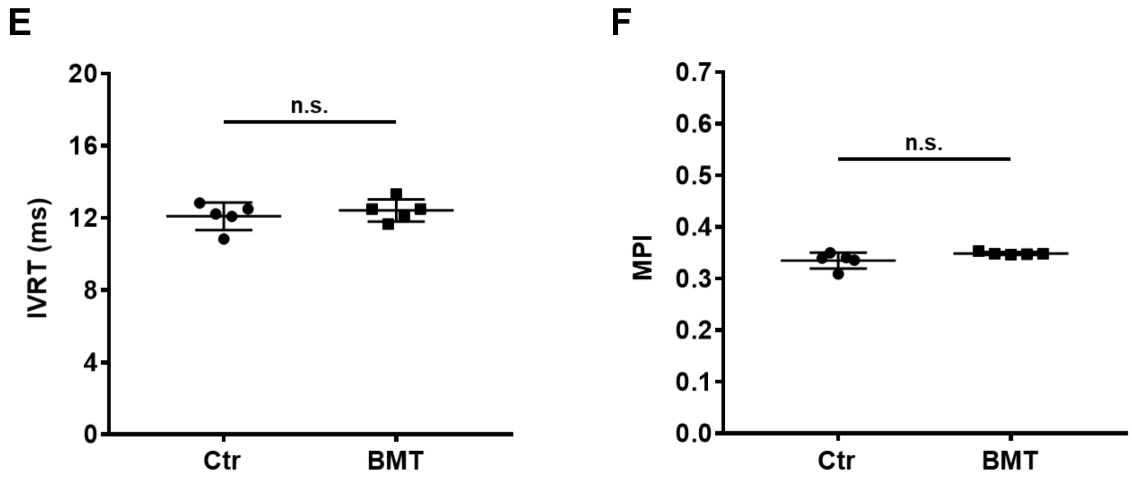

Supplemental Figure 2. Bone marrow transplant (BMT) does not affect cardiac function. A-F, Echo data show the difference between Ctr and BMT group mice in LVID;s, LV Vol.;s, LV Mass, IVCT, IVRT, and MPI. N=5 mice/group. n.s. indicates not statistically significant by the GraphPad *t* test. LVID;s, left ventricular internal diameter in systole; LV Vol.;s, left ventricular volume in systole; LV mass, left ventricular mass; IVCT, isovolumic contraction time; IVRT, isovolumic relaxation time; MPI, myocardial performance index; Ctr, control; BMT, bone marrow transplant.

Supplemental Figure 3

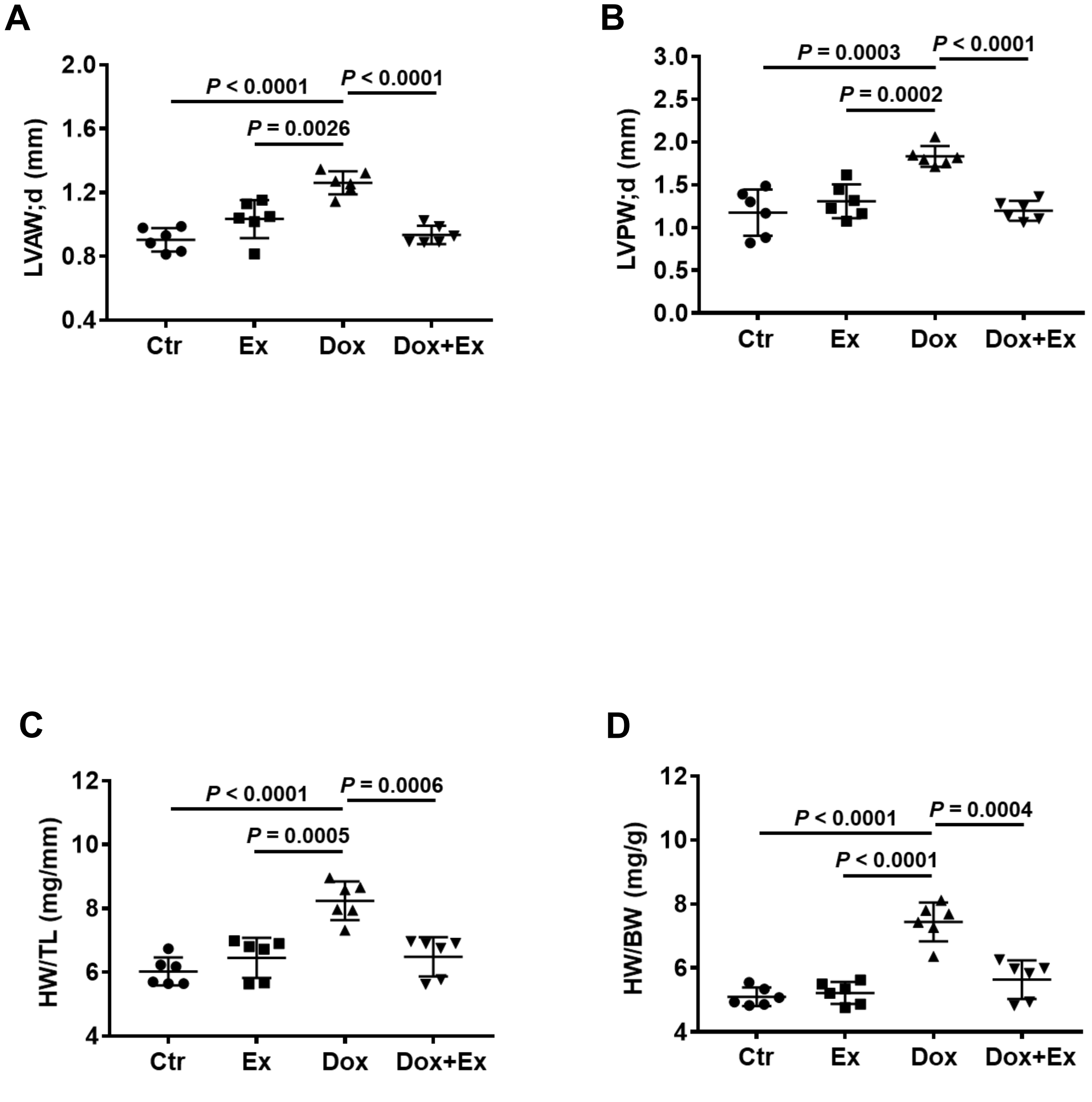

Supplemental Figure 3 (continued)

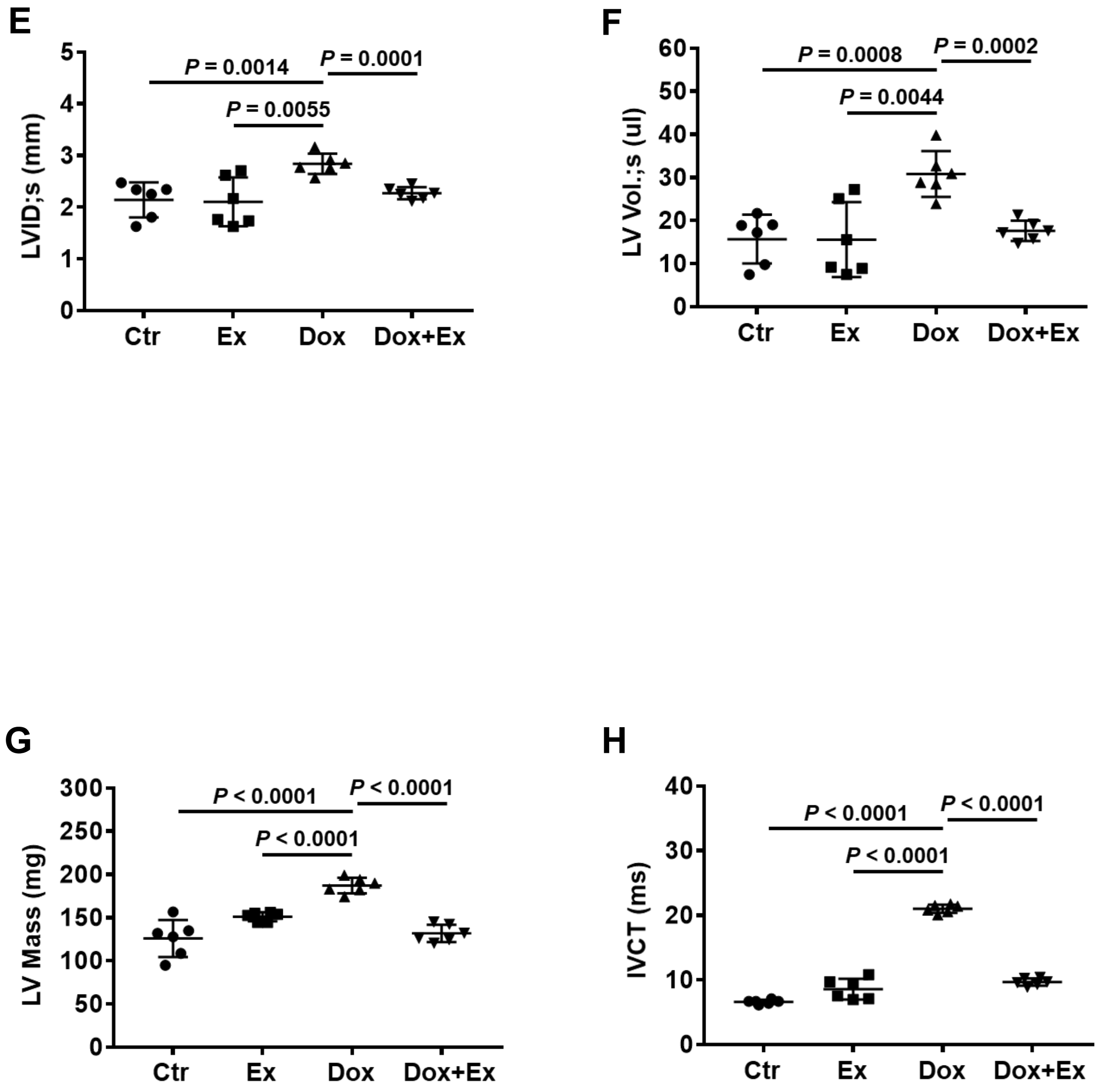

Supplemental Figure 3 (continued)

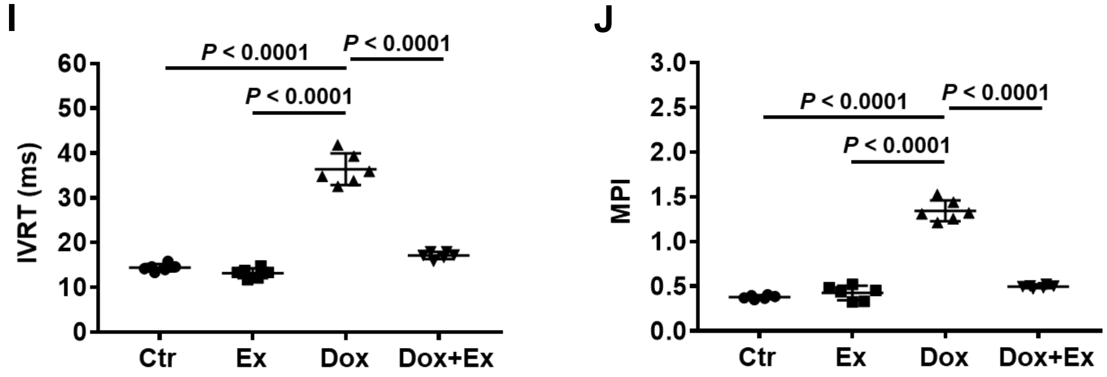

Supplemental Figure 3. Exercise preserves cardiac function after Dox treatment. **A-J,** Echo data show differences among Ctr, Ex, Dox and Dox+Ex group mice after treatment in LVAW;d, LVPW;d, HW/TL, HW/BW, LVID;s, LV Vol.;s, LV Mass, IVCT, IVRT, and MPI. N=6 mice/group. *P* values are indicated by the GraphPad *t* test. LVAW;d, left ventricular anterior wall thickness in diastole; LVPW;d, left ventricular posterior wall thickness in diastole; HW/TL, heart weight/tibia length; HW/BW, heart weight/body weight; LVID;s, left ventricular internal diameter in systole; LV Vol.;s, left ventricular volume in systole; LV Mass, left ventricular mass; IVCT, isovolumic contraction time; IVRT, isovolumic relaxation time; MPI, myocardial performance index.

Supplemental Figure 4

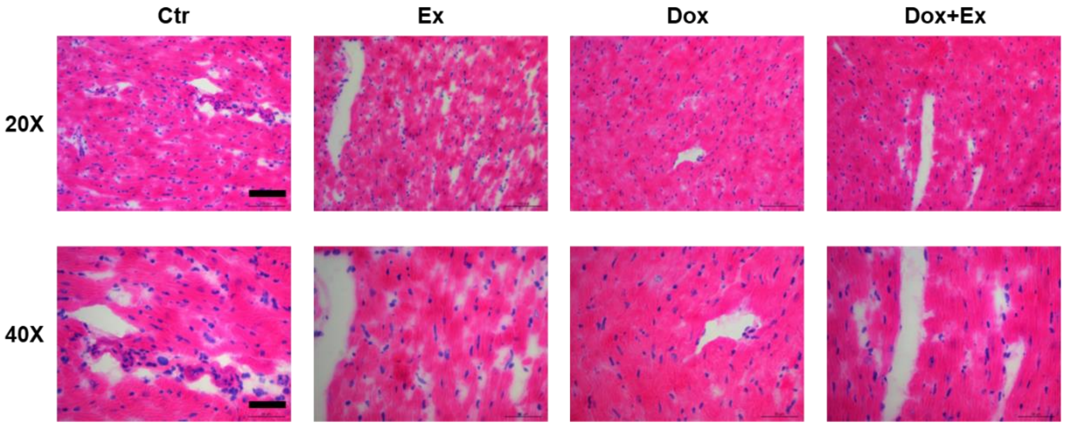

Supplemental Figure 4. Representative H&E staining images of heart sections from Ctr, Ex, Dox and Dox+Ex group mice. Magnification, 20x (top panel) and 40x (bottom panel); Scale bar, 100 μm (top panel) and 50 μm (bottom panel).

Supplemental Figure 5

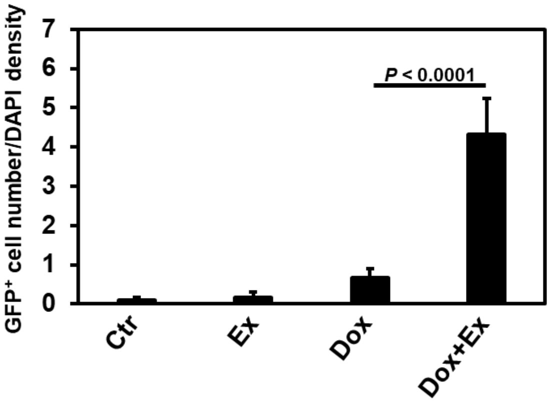

Supplemental Figure 5. Quantification of GFP positive cells in heart tissues normalized to DAPI density from Ctr, Ex, Dox and Dox+Ex group mice. N=5 mice/group, 3 slides/mouse. Values represent mean±SEM. *P* values are indicated by the Student's *t* test.

Supplemental Figure 6

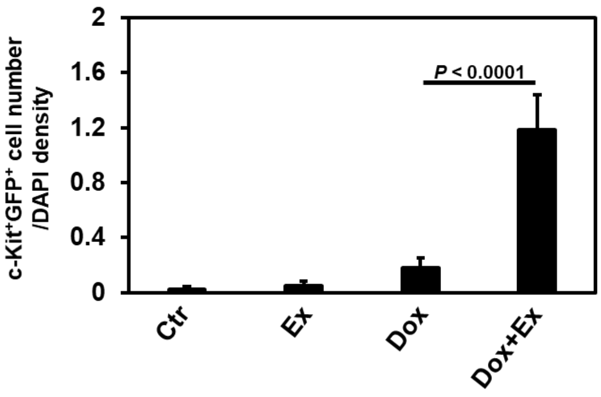

Supplemental Figure 6. Quantification of c-Kit and GFP double positive cells in heart tissues normalized to DAPI density from Ctr, Ex, Dox and Dox+Ex group mice. N=5 mice/group, 3 slides/mouse. Values represent mean±SEM. *P* values are indicated by the Student's *t* test.

Supplemental Figure 7

A

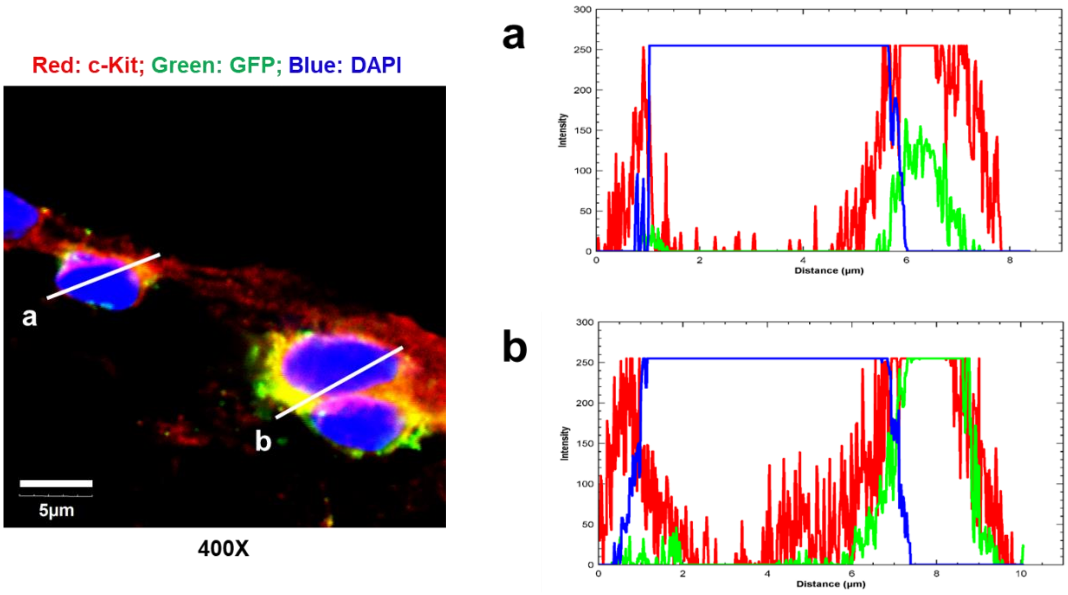

B

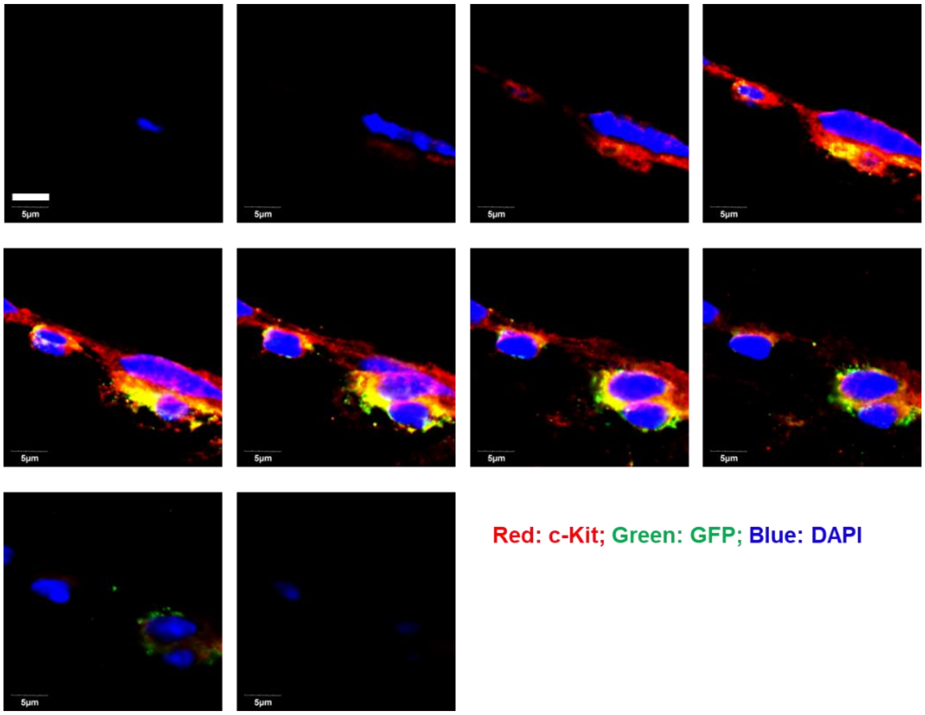

Supplemental Figure 7 (continued)

C

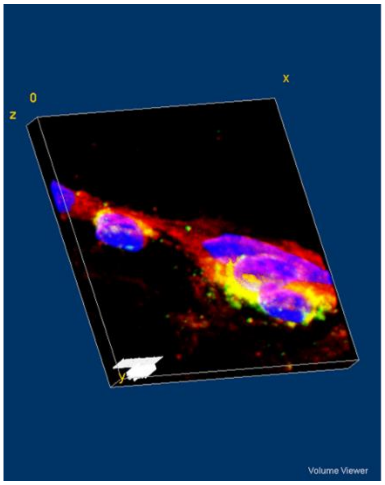

Red: c-Kit
Green: GFP
Blue: DAPI

D

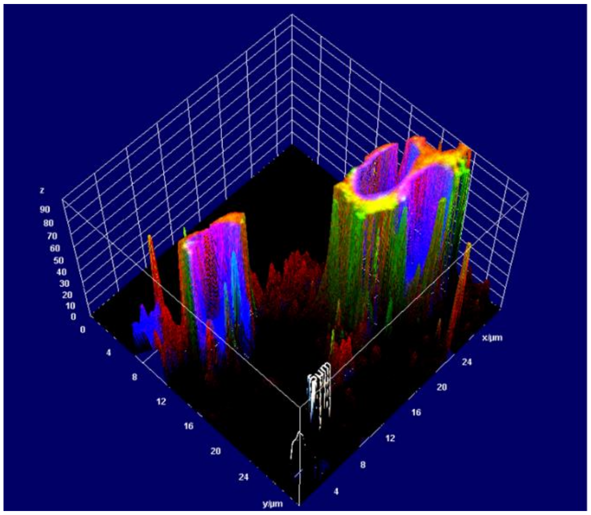

Red: c-Kit
Green: GFP
Blue: DAPI

Supplemental Figure 7. High magnification, Z-stack and 3D image analyses of c-Kit and GFP double positive cells in heart tissues from Dox+Ex group mice. A, A representative immunofluorescence image of a heart section stained for DAPI (blue), GFP (green) and c-Kit (red) from a Dox+Ex group mouse. Intensity of signals in lines a and b are monitored by ImageJ software. Magnification, 400x; Scale bar, 5 µm. **B,** Z-stack images of c-Kit and GFP double positive cells in (**A**) in heart tissue from a Dox+Ex group mouse captured by a FV1000 confocal laser scanning microscope. **C** and **D,** Z-stack images are reconstituted into 3D volume viewer (**C**) and 3D surface plot (**D**) by ImageJ software. Blue, DAPI; Green, GFP; Red, c-Kit. Magnification, 400x; Scale bar, 5 µm.

Supplemental Figure 8

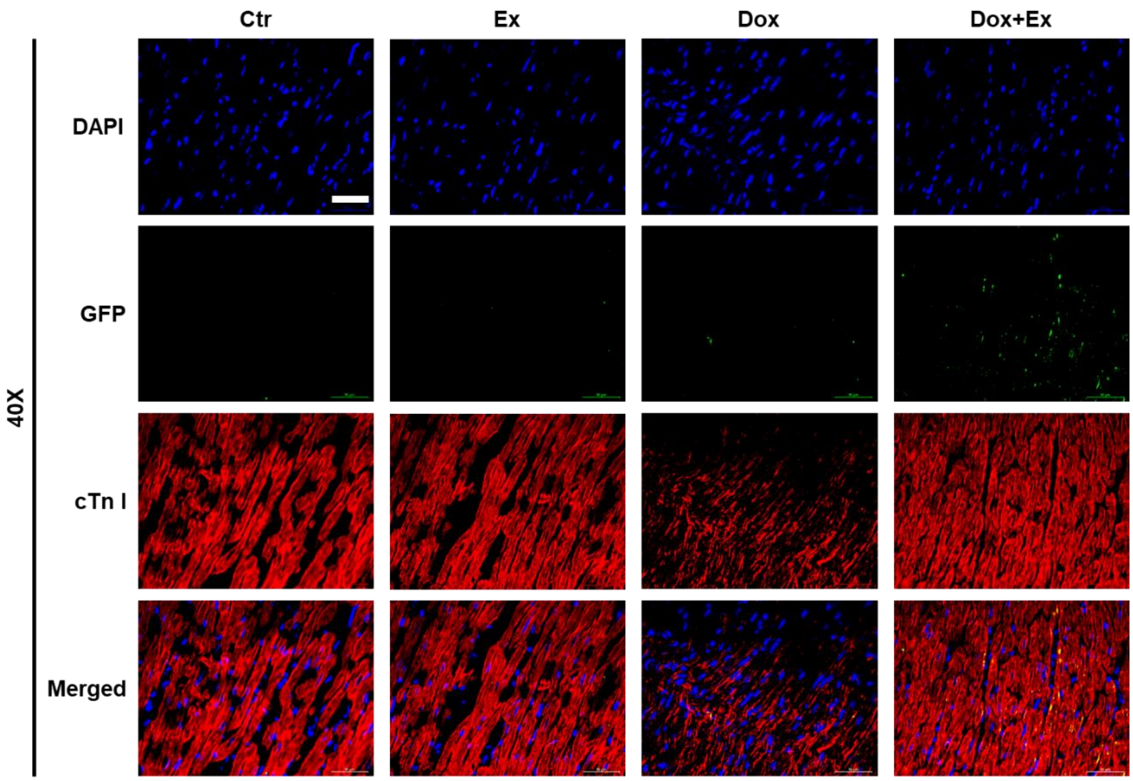

Supplemental Figure 8. Representative immunofluorescence images of heart sections stained for DAPI (blue), GFP (green) and cTn I (red) from Ctr, Ex, Dox and Dox+Ex group mice. Magnification, 40x; Scale bar, 50 µm.

Supplemental Figure 9

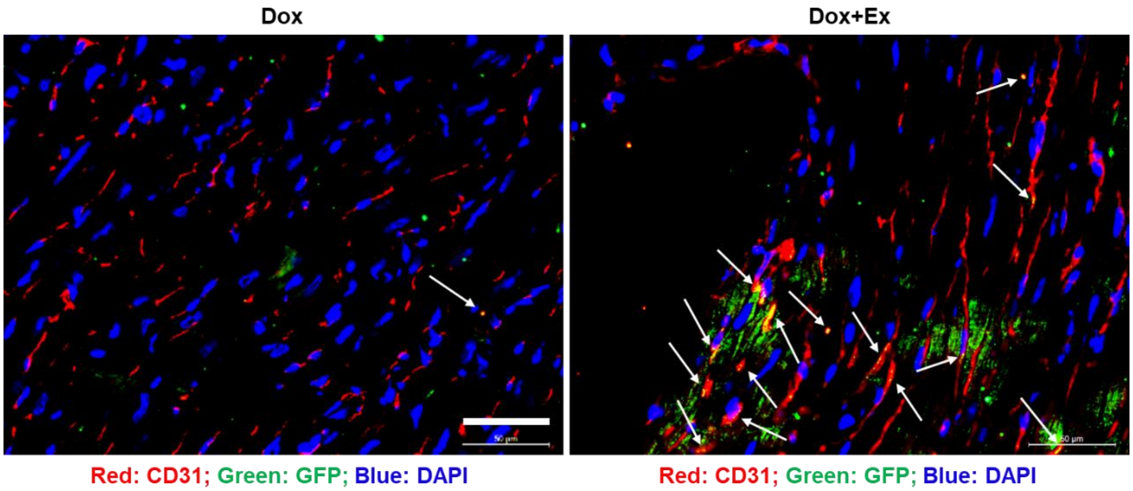

Supplemental Figure 9. Enlarged representative immunofluorescence merged images of heart sections stained for DAPI (blue), GFP (green) and CD31 (red) from Dox and Dox+Ex group mice shown in Figure 5C. Arrows indicate co-localization (yellow) of GFP (green) and CD31 (red). Magnification, 40x; Scale bar, 50 µm.

Supplemental Figure 10

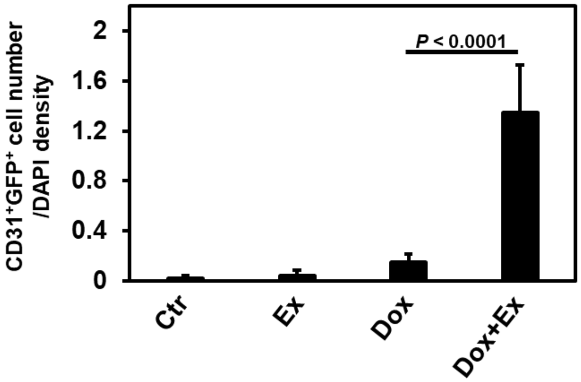

Supplemental Figure 10. Quantification of CD31 and GFP double positive cells in heart tissues normalized to DAPI density from Ctr, Ex, Dox and Dox+Ex group mice. N=5 mice/group, 3 slides/mouse. Values represent mean±SEM. *P* values are indicated by the Student's *t* test.

Supplemental Figure 11

A

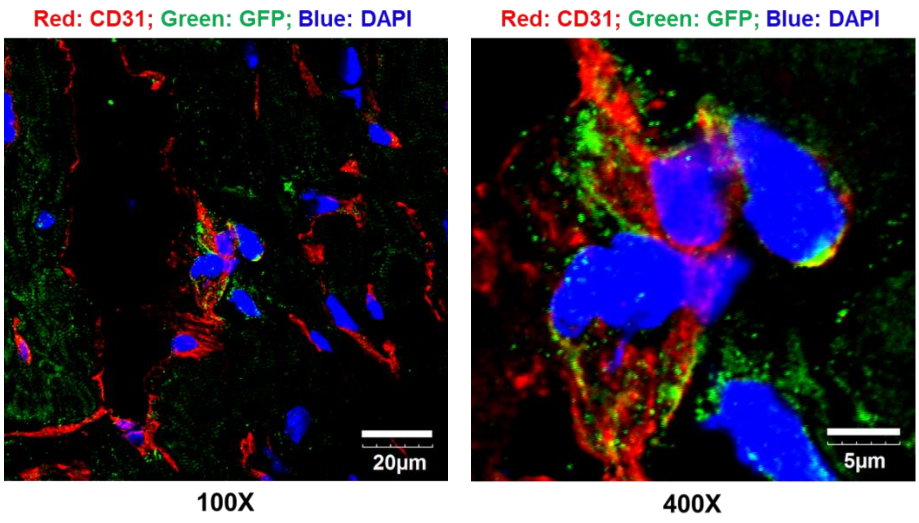

B

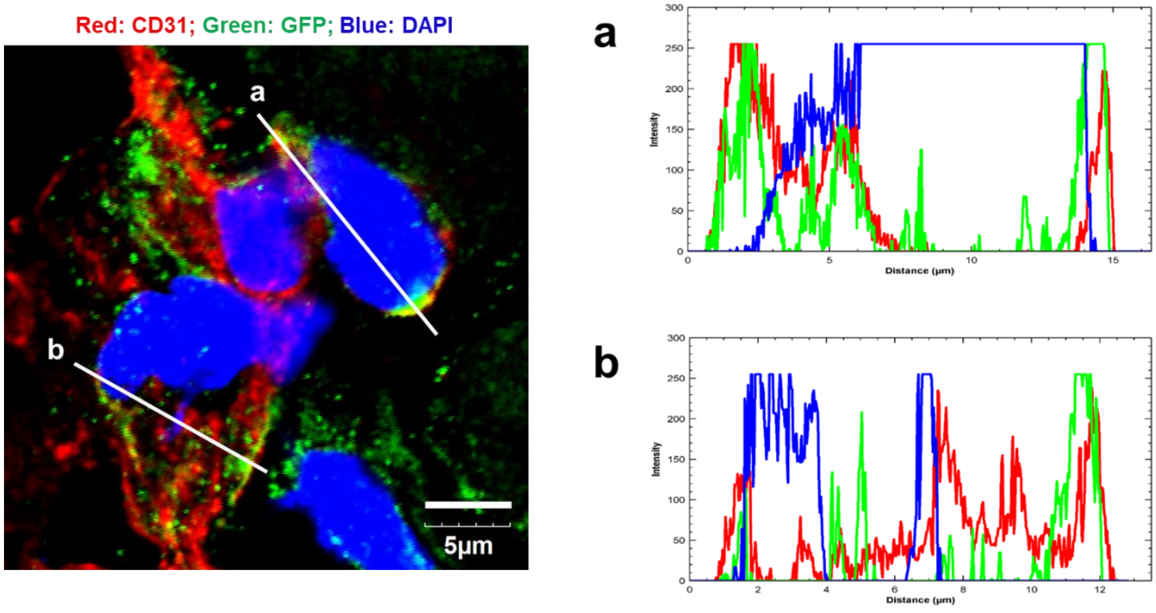

Supplemental Figure 11 (continued)

C

Red: CD31; Green: GFP; Blue: DAPI

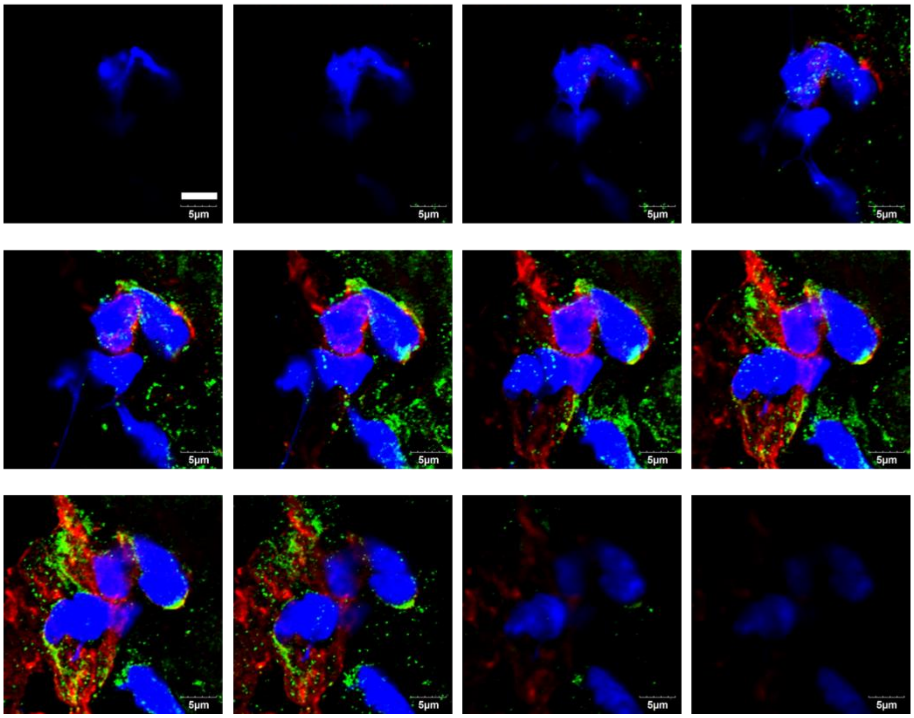

D

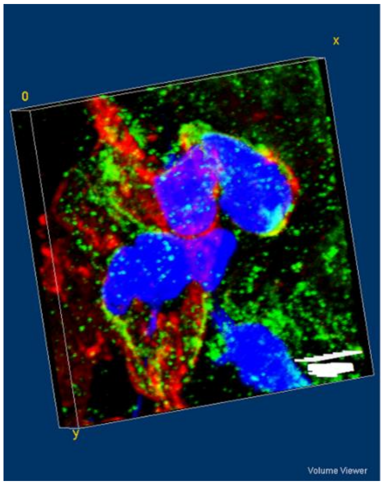

Red: CD31
Green: GFP
Blue: DAPI

E

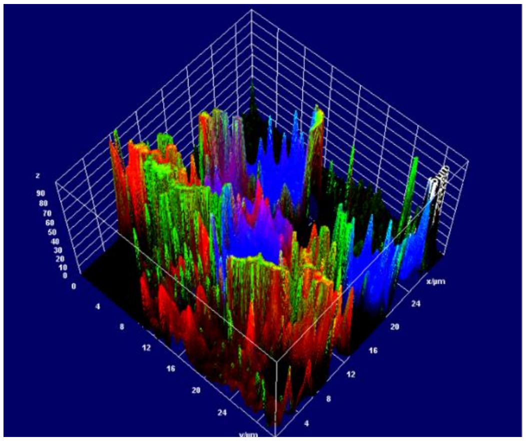

Red: CD31
Green: GFP
Blue: DAPI

Supplemental Figure 11. High magnification, Z-stack and 3D image analyses of CD31 and GFP double positive cells in heart tissues from Dox+Ex group mice. A, A representative immunofluorescence image of heart section stained for DAPI (blue), GFP (green) and CD31 (red) from a Dox+Ex group mouse under magnification 100x (left) and 400x (right). Scale bar, 20 µm (left) and 5 µm (right). **B,** Intensity of signals in lines a and b in (**A**) (right) are monitored by ImageJ software. **C,** Z-stack images of CD31 and GFP double positive cells in (**A**) (right) in heart tissue from a Dox+Ex group mouse captured by a FV1000 confocal laser scanning microscope. **D** and **E,** Z-stack images are reconstituted into 3D volume viewer (**D**) and 3D surface plot (**E**) by ImageJ software. Blue, DAPI; Green, GFP; Red, CD31. Magnification, 400x; Scale bar, 5 µm.

Supplemental Figure 12

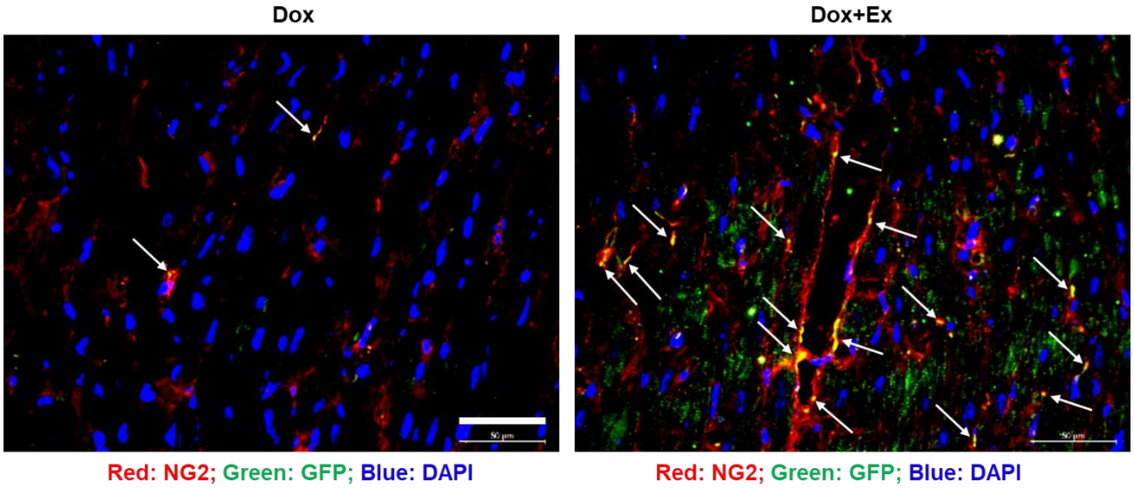

Supplemental Figure 12. Enlarged representative immunofluorescence merged images of heart sections stained for DAPI (blue), GFP (green) and NG2 (red) from Dox and Dox+Ex group mice shown in Figure 6C. Arrows indicate co-localization (yellow) of GFP (green) and NG2 (red). Magnification, 40x; Scale bar, 50 µm.

Supplemental Figure 13

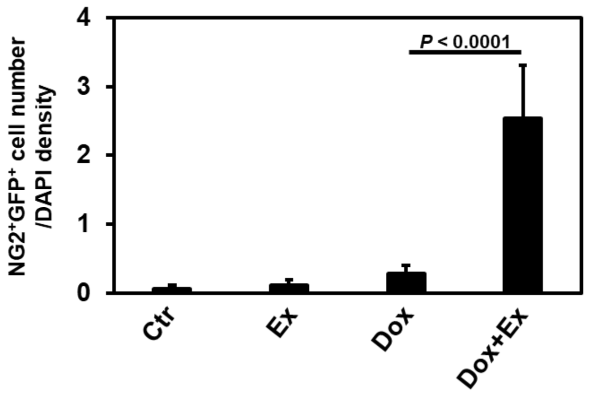

Supplemental Figure 13. Quantification of NG2 and GFP double positive cells in heart tissues normalized to DAPI density from Ctr, Ex, Dox and Dox+Ex group mice. N=5 mice/group, 3 slides/mouse. Values represent mean±SEM. *P* values are indicated by the Student's *t* test.

Supplemental Figure 14

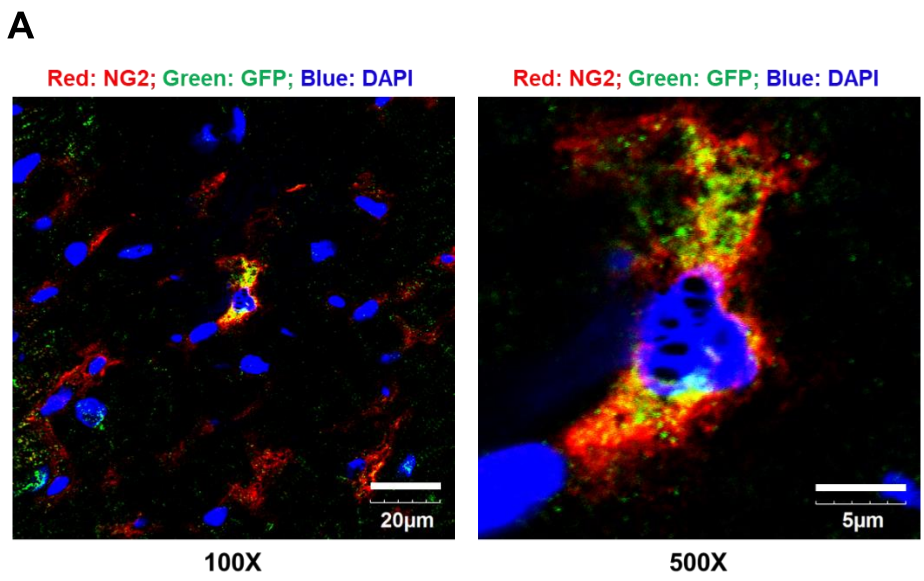

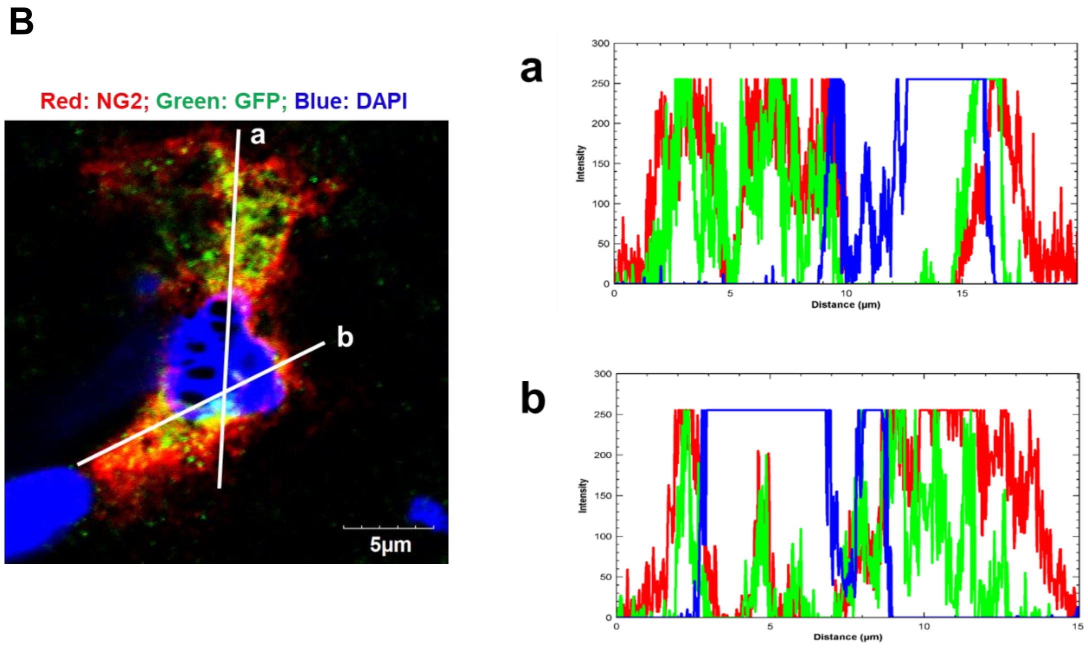

Supplemental Figure 14 (continued)

C

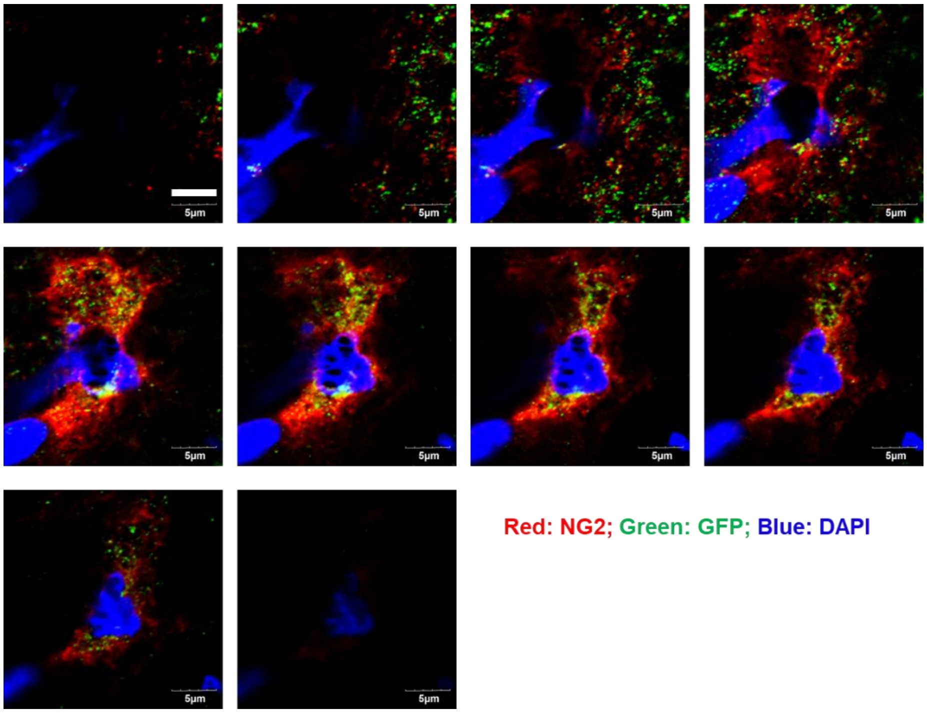

Red: NG2; Green: GFP; Blue: DAPI

D

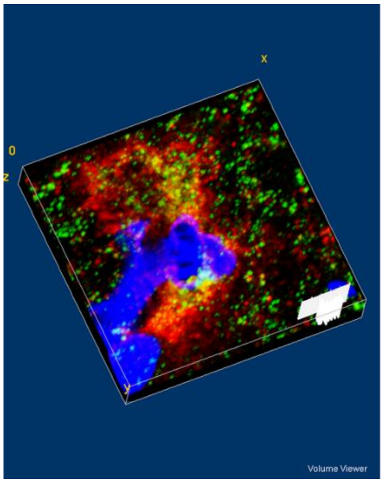

Red: NG2
Green: GFP
Blue: DAPI

E

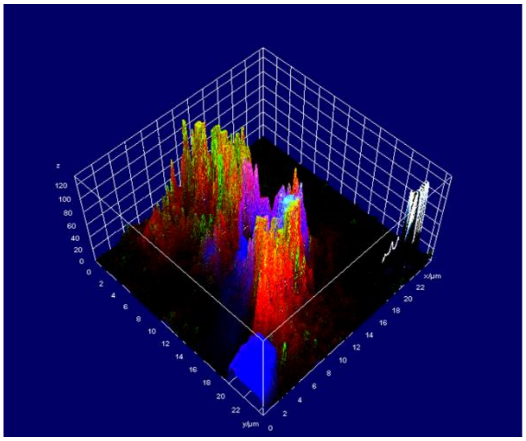

Red: NG2
Green: GFP
Blue: DAPI

Supplemental Figure 14. High magnification, Z-stack and 3D image analyses of NG2 and GFP double positive cells in heart tissues from Dox+Ex group mice. A, A representative immunofluorescence image of heart section stained for DAPI (blue), GFP (green) and NG2 (red) from a Dox+Ex group mouse under magnification 100x (left) and 500x (right). Scale bar, 20 μm (left) and 5 μm (right). **B,** Intensity of signals in lines a and b in (**A**) (right) are monitored by ImageJ software. **C,** Z-stack images of NG2 and GFP double positive cells in (**A**) (right) in heart tissue from a Dox+Ex group mouse captured by a FV1000 confocal laser scanning microscope. **D** and **E,** Z-stack images are reconstituted into 3D volume viewer (**D**) and 3D surface plot (**E**) by ImageJ software. Blue, DAPI; Green, GFP; Red, NG2. Magnification, 500x; Scale bar, 5 μm.

Supplemental Figure 15

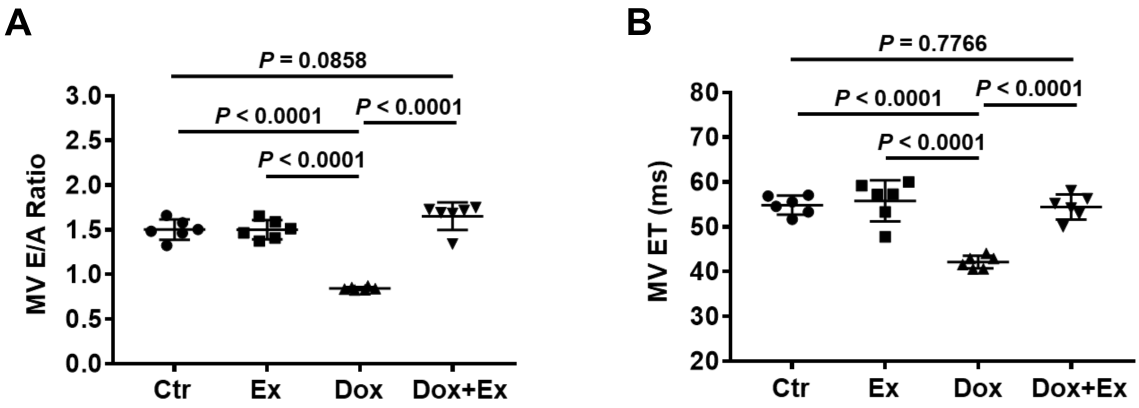

Supplemental Figure 15. Exercise prevents Dox-induced decrease in cardiac blood flow in transplanted mice. **A** and **B,** Echo data show differences among Ctr, Ex, Dox and Dox+Ex group mice after treatment in MV E/A Ratio and MV ET. N=6 mice/group. *P* values are indicated by the GraphPad *t* test. Ctr vs Dox+Ex, not statistically significant. MV E/A Ratio, mitral valve peak E and A velocity ratio; MV ET, mitral valve ejection time.
