# Supplementary material for: Exercise Inhibits Doxorubicin-Induced Damage to Cardiac Vessels and Activation of Hippo/YAP-Mediated Apoptosis
Source: Cancers (Basel). 2021 Jun 1;13(11):2740. doi: 10.3390/cancers13112740 (PMC8198139; doi:10.3390/cancers13112740)
Supplement: Supplementary file 1 [file cancers-13-02740-s001.zip › Supplemental Methods_Tables_Videos.pdf]

SUPPLEMENTAL MATERIAL

Supplemental Methods

Mice

All mouse procedures were performed according to institutional guidelines and governmental regulations. All mouse experimental procedures were approved by the Institutional Animal Care and Use Committee (IACUC) at The University of Texas MD Anderson Cancer Center under protocol number IACUC00001615-RN00. Recipient mice of strain C57BL/6 were purchased at 4 weeks old from the Animal Facility, Experimental Radiation Oncology, MD Anderson Cancer Center, Houston, TX. Donor GFP transgenic mice of strain C57BL/6-Tg(UBC-GFP)30Scha/J were purchased from The Jackson Laboratory, Bar Harbor, ME.

Cell Culture

Human Cardiac Myocytes (HCMs) were cultured with Cardiac Myocyte Medium (CMM) in Poly-L-Lysine-coated dishes and supplemented with 5% Fetal Bovine Serum (FBS), 1% Cardiac Myocyte Growth Supplement (CMGS) and 1% Penicillin/Streptomycin solution (P/S) (all were purchased from ScienCell Research Laboratories, Inc., Carlsbad, CA). Cells were maintained in an incubator with 5% CO2 atmosphere at 37°C. Doxorubicin (Dox) was obtained from Teva Parenteral Medicines, Inc. (NDC 0703-5046-01; Irvine, CA). Verteporfin (VP) was purchased from Sigma-Aldrich (Cat. #SML0534).

Bone Marrow Transplant (BMT)

Whole bone marrow (BM) cells were collected by flushing femurs from donor GFP transgenic mice with phosphate-buffered saline (PBS). Recipient C57BL/6 mice were treated intraperitoneally with 25 mg/kg of Busulfan (Cat. #B2635; Sigma) four days before BMT and with 30 µg of anti-CD4 (Clone GK1.5; Cat. #BE0003-1; Bio-X-Cell) and anti-CD8 (Clone 2.43; Cat. #BE0061; Bio-X-Cell) antibodies two days before BMT. Treated mice were then injected intravenously with 5×10^6 GFP positive BM cells. After allowing four weeks for BM cell engraftment, the transplant efficiency was confirmed by evaluating GFP positive BM cells collected from at least three representative transplanted mice using Flow Cytometry (BD FACSCalibur; BD Biosciences; Figure S1).

Exercise (Ex) and Doxorubicin (Dox) Treatment

Treadmill Exercise Protocol: Mice were exercised 5 or 6 days a week with 1 or 2 day off for a total of 2 weeks (total 10 or 12 days of exercise) as indicated. Mice were placed in a treadmill with an operating protocol consisting of 45 minutes of constant walking at a rate of 12 m/min at 0% slope with 2 minutes of ramp up from 0 m/ min and 2 minutes of cool down to 0 m/min at the beginning and end of exercise respectively as reported previously [43]. When exercise was completed, the mice were immediately returned to their cages and allowed to act freely.

Doxorubicin Treatment: Mice were injected intravenously with 2.5 mg/kg of Dox, twice a week for 2 weeks. Dox injection was administered on days 3, 5, 10 and 12 after the start of the exercise protocol, as illustrated in figure 1A and 3A.

Echocardiographic Imaging

Before and after bone marrow transplantation and Dox/Ex treatment, mouse cardiac function and morphology were monitored under anesthetization with 1% isoflurane when the heart rate reached around 425 bpm using a Vevo 2100 High Resolution Imaging System equipped with a 30-MHz probe (MS400) (FUJIFILM VisualSonics, Inc., Toronto, Canada) as described previously [18, 43]. The parasternal long axis view of mouse heart under B-mode was obtained by adjusting the angle of the transducer and focusing depth to visualize the left ventricle, the intraventricular septal wall, and a slight portion of the right ventricular wall. Next, the mode was switched to M-mode and the M-mode cursor was placed perpendicular to the LV walls at the level of the papillary muscle to acquire images. The parasternal short axis view of mouse heart in B-mode was achieved by rotating the transducer 90° clockwise from the parasternal long axis view and adjusting the transducer to give a horizontal cross-sectional "transverse" view of the heart with both papillary muscles clearly visible and located to the right (the 2 o'clock and 4 o'clock positions). After switching to M-mode, place the M-mode axis at the mid-level of the left ventricle to acquire images. The apical four-chamber view of both the left and right ventricles with the atria at the bottom of the heart was achieved by tilting the upper left corner of the platform to angle the mouse's head down and orienting the transducer towards the right shoulder of the mouse from the short axis view in B-mode. When the mitral valve in B-mode was observed, switch to color Doppler mode and place the sample volume at the tip of the mitral valve. And then switch to PW Doppler mode to assess flow patterns across the mitral valve by aligning the Doppler probe cursor parallel to the direction of mitral blood flow and using a probe angle less

than 20° to determine peak velocity. The aortic arch view of the aorta and major arterial branches in B-mode was achieved by turning the mouse into the left decubitus position through tilting up the left side of the platform. With the notch towards the chin of the mouse, put the ultrasound transducer on the chest along the right parasternal line. Then tilt up the transducer at the level of the scapula followed by turning slightly clockwise to see the aortic arch. Switch to color Doppler mode to examine the directionality and velocity of aortic blood flow followed by switching to pulse wave (PW) Doppler mode and placing sample volume to get waveforms of aortic blood flow and determine peak velocity. Frame rate for all modes was set to 300 frames per minute. And all mode images were captured at least 3 times for each mouse. Mouse echocardiographic functional and morphological parameters were measured and analyzed using the parasternal long axis or short axis M-mode and Doppler imaging with Vevo LAB 3.1.1 software provided by the system's manufacturer (FUJIFILM VisualSonics, Inc., Toronto, Canada) and GraphPad Prism 6 software (GraphPad Software, Inc.).

Immunohistochemical Analysis

Frozen slides and H&E staining were carried out by the MD Anderson Research Histology Core Laboratory. Fixation and permeabilization of frozen tissues were performed by submersion in cold acetone for 10 minutes as described previously [38]. The slides were rinsed with PBS and blocked in 4% fish gelatin blocking agent (Biotium) diluted in PBS at room temperature (RT) for 1 hour. Then tissues were incubated with the following primary antibodies diluted in 4% fish gel at 4°C overnight: rabbit anti-GFP, goat anti-GFP, rabbit anti-Cardiac Troponin I (cTn I) and rabbit anti-Vimentin (Abcam);

rat anti-CD31 (BD Pharmingen); mouse anti-NG2 (Santa Cruz Biotechnology); goat anti-c-Kit (R&D Systems); rabbit anti-phospho-YAP (Ser127), and rabbit anti-Cleaved Caspase-3 (Cell Signaling Technology). Tissues were washed with PBS and then incubated with the following secondary fluorescent antibodies diluted in 4% fish gel at RT for 2 hours: Alexa Fluor 488-conjugated anti-rabbit and anti-goat; Alexa Fluor 647-conjugated anti-rabbit, anti-rat, anti-mouse, and anti-goat (Abcam). Finally, tissues were washed with PBS and coverslips were mounted using Fluoro-Gel II with DAPI (Electron Microscopy Sciences). Images were captured with a Leica DM5500 B upright microscope imaging system (Leica Microsystems) and analyzed using Adobe Photoshop software.

Confocal Laser Scanning Microscope

The high magnification imaging and Z-series stack were performed in the Flow Cytometry & Cellular Imaging Facility at MD Anderson Cancer Center. Stained slides were visualized under a FV1000 confocal laser scanning microscope with ISS upgrade (Olympus Corp.; Shinjuku, Tokyo, Japan) and images were captured using FV10-ASW 4.0 software (Olympus Corp.; Shinjuku, Tokyo, Japan). Images were acquired at a resolution of 1024 x 1024 pixels using an Apo100xOHR numerical aperture (NA) 1.65 oil objective lens at 1x, 2.5x, 3x, 4x, or 5x zoom respectively according to the size of cells of interest as shown in figures. Samples were excited using a laser at LD 405 nm for DAPI, Argon 488 nm for Alex Fluor 488, LD 559 nm for Alex Fluor 594, and LD 635 nm for Alex Fluor 647. A Z-series stack with step-size set as Optimal (Op.) was collected and processed into a single Z-projection. Images were acquired sequentially

to minimize cross contamination from multiple emission spectra. Identical settings for laser intensity and other image capture parameters were applied for comparison of staining from different mouse groups [37, 38]. Examination of signal intensity and reconstitution of Z-series stacks into 3D volume viewer, 3D surface plot and videos were performed using ImageJ software (National Institutes of Health, Bethesda, MD).

MTT Assay

Human Cardiac Myocytes (HCMs) were cultured in 96-well microplates and treated with various concentrations of Dox for the time periods indicated in the figures. After labeling with 3-(4,5-Dimethyl-2-thiazolyl)-2,5-diphenyl-2H-tetrazolium bromide (MTT/thiazolyl blue tetrazolium bromide; Sigma), cell growth was monitored using a SpectraMax Plus 384 microplate reader (Molecular Devices, LLC). Data were analyzed using Excel [9].

Western Blot Analysis

Treated Human Cardiac Myocytes (HCMs) and mouse heart tissues were collected and cell extracts were prepared for Western blot analysis with the indicated antibodies under conditions as described in previous reports [35, 36]. Briefly, cell extracts were prepared by incubation in Cell Lysis Buffer (Cell Signaling Technology, Danvers, MA) plus protease and phosphatase inhibitors (Thermo Scientific) for 30 minutes on ice. The lysates were sonicated and clarified by centrifugation at 13,000 g for 10 minutes at 4°C, and the supernatants were collected and boiled in sodium dodecyl sulfate (SDS) loading buffer (Cell Signaling Technology, Danvers, MA). Proteins were separated by electrophoresis on 10% SDS-polyacrylamide gels (Bio-Rad Laboratories, Hercules,

CA), transferred to nitrocellulose membranes (Whatman Schleicher & Schuell, Keene, NH), and subjected to Western blotting by incubating the membranes with the following primary antibodies at 4°C overnight: rabbit anti-MST1, anti-LATS1, anti-phospho-LATS1 (Thr1079), anti-YAP, anti-phospho-YAP (Ser127), anti-Cleaved Caspase-3 (Asp175), anti-Cleaved PARP (Asp214), anti-PARP (all from Cell Signaling Technology); rabbit anti-phospho-Mst1/2 (pThr183) (Sigma); and rabbit anti-CTGF, anti-CYR61, anti-Histone H3 (Abcam). After washing, the membranes were incubated with the corresponding HRP-conjugated goat anti-rabbit secondary antibody (Thermo Scientific) at RT for 2 hours. Protein bands were developed using SuperSignal West Dura Extended Duration Substrate (Thermo Scientific), detected using a Kodak Medical X-Ray Processor 104 (Eastman Kodak Company, Rochester, NY) or a ChemiDoc Touch Imaging System (BIO-RAD), and analyzed using Adobe Photoshop software.

Statistical Analysis

Statistical analysis of echocardiographic data was performed using the GraphPad *t* test. For other data analysis, Student’s *t* test was applied. Data are presented as mean±SEM, and $P<0.05$ was considered statistically significant.

Supplemental Tables

Supplemental Table 1. List of antibodies used in Western blot analysis.

Antibody	Manufacture	Catalog #	Host Species	Dilution	Application
MST1	Cell Signaling Technology	3682S	Rabbit	1:800	WB
LATS1	Cell Signaling Technology	3477S	Rabbit	1:800	WB
Phospho-LATS1 (Thr1079)	Cell Signaling Technology	8654S	Rabbit	1:800	WB
YAP	Cell Signaling Technology	14074S	Rabbit	1:800	WB
Phospho-YAP (Ser127)	Cell Signaling Technology	13008S	Rabbit	1:800	WB
Cleaved Caspase-3 (Asp175)	Cell Signaling Technology	9664S	Rabbit	1:1000	WB
Cleaved PARP (Asp214)	Cell Signaling Technology	5625S	Rabbit	1:1000	WB
PARP	Cell Signaling Technology	9542S	Rabbit	1:1000	WB
Phospho-Mst1/2 (pThr183)	Sigma	SAB4504042	Rabbit	1:800	WB
CTGF	Abcam	ab6992	Rabbit	1:800	WB
CYR61	Abcam	ab228592	Rabbit	1:800	WB
Histone H3	Abcam	ab1791	Rabbit	1:8000	WB
Anti-rabbit-HRP	Thermo Scientific	32460	Goat	1:4000	WB

Supplemental Table 2. List of antibodies used in immunohistochemical analysis.

Antibody	Manufacture	Catalog #	Host Species	Dilution	Application
GFP	Abcam	ab6556	Rabbit	1:300	IF
GFP	Abcam	ab5450	Goat	1:300	IF
Cardiac Troponin I (cTn I)	Abcam	ab47003	Rabbit	1:300	IF
Vimentin	Abcam	ab92547	Rabbit	1:300	IF
CD31	BD Pharmingen	553370	Rat	1:450	IF
NG2	Santa Cruz Biotechnology	sc-33666	Mouse	1:150	IF
c-Kit	R&D Systems	AF1356	Goat	1:200	IF
Phospho-YAP (Ser127)	Cell Signaling Technology	13008S	Rabbit	1:300	IF
Cleaved Caspase-3 (Asp175)	Cell Signaling Technology	9664S	Rabbit	1:300	IF
Alexa Fluor 488-conjugated anti-rabbit	Abcam	ab150073	Donkey	1:500	IF
Alexa Fluor 488-conjugated anti-goat	Abcam	ab150129	Donkey	1:500	IF
Alexa Fluor 647-conjugated anti-rabbit	Abcam	ab150075	Donkey	1:400	IF
Alexa Fluor 647-conjugated anti-rat	Abcam	ab150155	Donkey	1:400	IF
Alexa Fluor 647-conjugated anti-mouse	Abcam	ab150107	Donkey	1:400	IF
Alexa Fluor 647-conjugated anti-goat	Abcam	ab150135	Donkey	1:400	IF

Supplemental Videos

Supplemental Video 1. Video of Z-stack images in Figure S7B reconstituted by ImageJ software. Blue, DAPI; Green, GFP; Red, c-Kit. Magnification, 400x; Scale bar, 5 µm.

Supplemental Video 2. Video of 3D surface plot of Z-stack images in Figure S7B reconstituted by ImageJ software. Blue, DAPI; Green, GFP; Red, c-Kit. Magnification, 400x; Scale bar, 5 µm.

Supplemental Video 3. Video of Z-stack images in Figure S11C reconstituted by ImageJ software. Blue, DAPI; Green, GFP; Red, CD31. Magnification, 400x; Scale bar, 5 µm.

Supplemental Video 4. Video of 3D surface plot of Z-stack images in Figure S11C reconstituted by ImageJ software. Blue, DAPI; Green, GFP; Red, CD31. Magnification, 400x; Scale bar, 5 µm.

Supplemental Video 5. Video of Z-stack images in Figure S14C reconstituted by ImageJ software. Blue, DAPI; Green, GFP; Red, NG2. Magnification, 500x; Scale bar, 5 µm.

Supplemental Video 6. Video of 3D surface plot of Z-stack images in Figure S14C reconstituted by ImageJ software. Blue, DAPI; Green, GFP; Red, NG2. Magnification, 500x; Scale bar, 5 µm.
