# Supplementary material for: Exercise Inhibits Doxorubicin-Induced Damage to Cardiac Vessels and Activation of Hippo/YAP-Mediated Apoptosis
Source: Cancers (Basel). 2021 Jun 1;13(11):2740. doi: 10.3390/cancers13112740 (PMC8198139; doi:10.3390/cancers13112740)
Supplement: Supplementary file 1 [file cancers-13-02740-s001.zip › Supplemental Western Blots.pdf]

SUPPLEMENTAL MATERIAL-Western Blot

Exercise Inhibits Doxorubicin-Induced Damage to Cardiac Vessels and Activation of Hippo/YAP-Mediated Apoptosis

Rong-Hua Tao*, MD, PhD; Masato Kobayashi, PhD; Yuanzheng Yang, PhD; Eugenie S. Kleinerman*, MD

***Correspondence to**

Dr. Eugenie S. Kleinerman, MD

Department of Pediatrics-Research,

Division of Pediatrics,

The University of Texas MD Anderson Cancer Center,

1515 Holcombe Boulevard, Unit #853, Houston, Texas 77030

Tel: (713)-792-8110

Fax: (713)-563-5407

Email: ekleiner@mdanderson.org

Dr. Rong-Hua Tao, MD & PhD

Department of Pediatrics-Research,

Division of Pediatrics,

The University of Texas MD Anderson Cancer Center,

1515 Holcombe Boulevard, Unit #853, Houston, Texas 77030

Tel: (713)-563-7333

Fax: (713)-563-5407

Email: rtao@mdanderson.org

Supplemental Western Blot

Fig. 3F

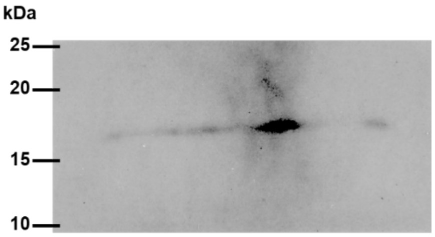

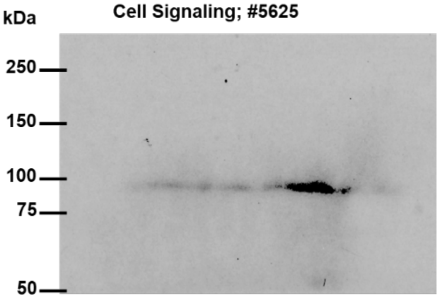

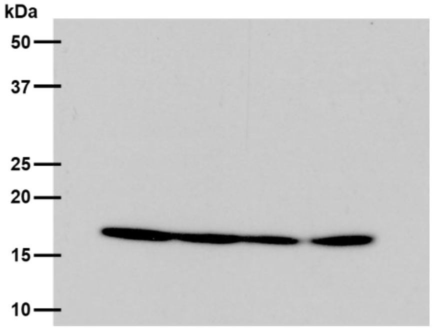

Fig. 8B

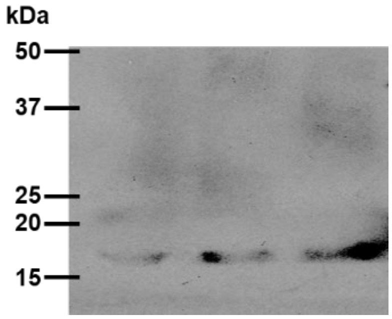
kDa
50
37
25
20
15

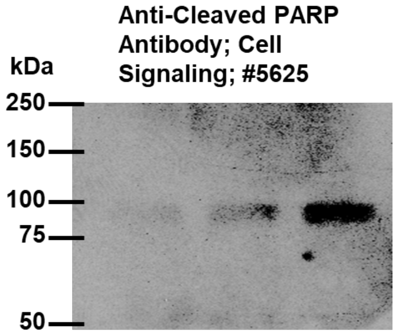
Anti-Cleaved PARP Antibody; Cell Signaling; #5625
kDa
250
150
100
75
50

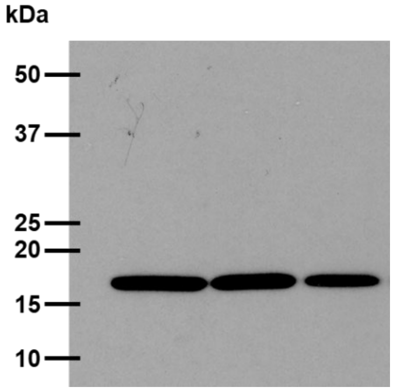
kDa
50
37
25
20
15
10

Fig. 8C

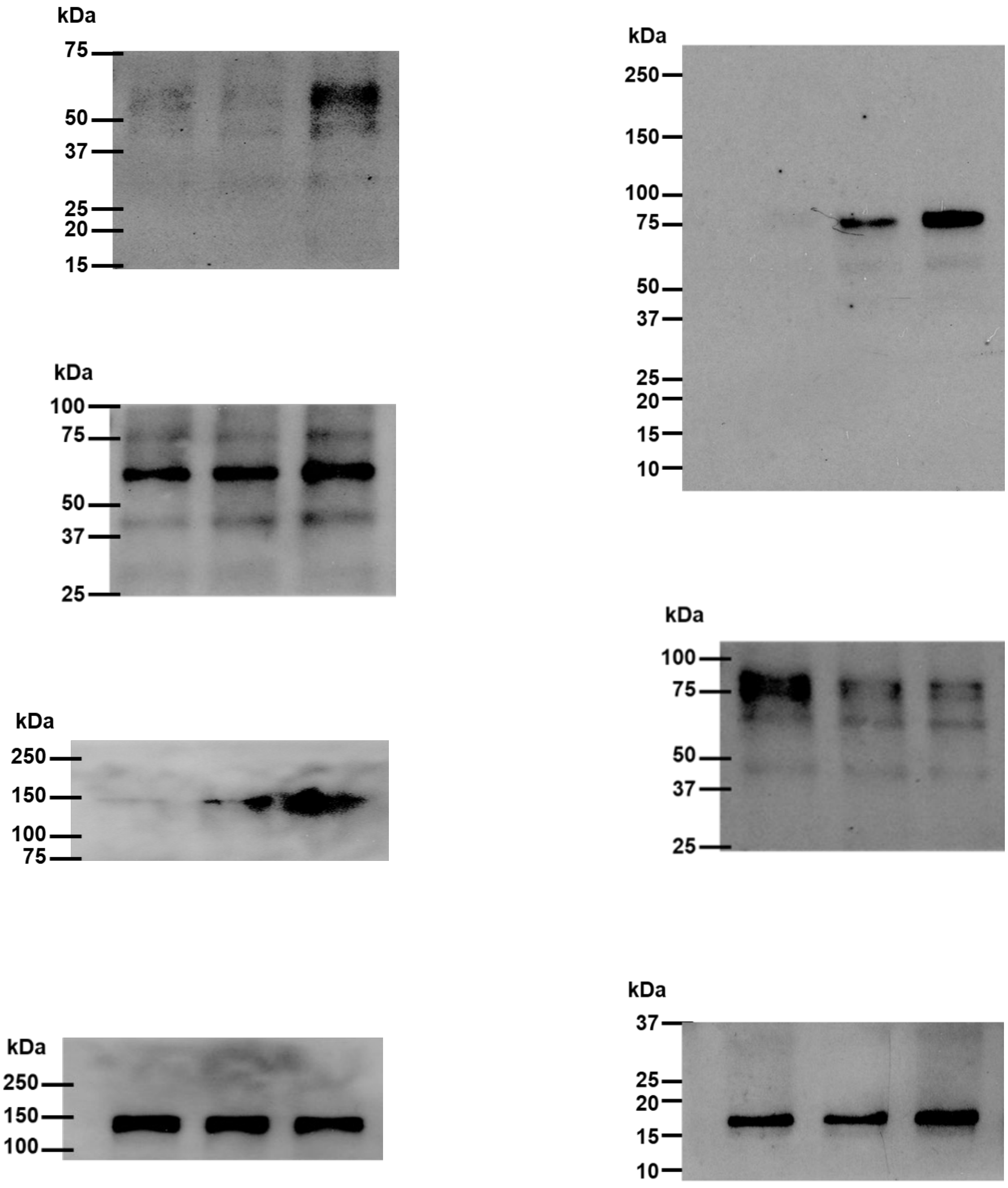
kDa
75
50
37
25
20
15
kDa
100
75
50
37
25
kDa
250
150
100
75
kDa
250
150
100
kDa
250
150
100
75
50
37
25
20
15
10
kDa
100
75
50
37
25
kDa
37
25
20
15
10

Fig. 8E

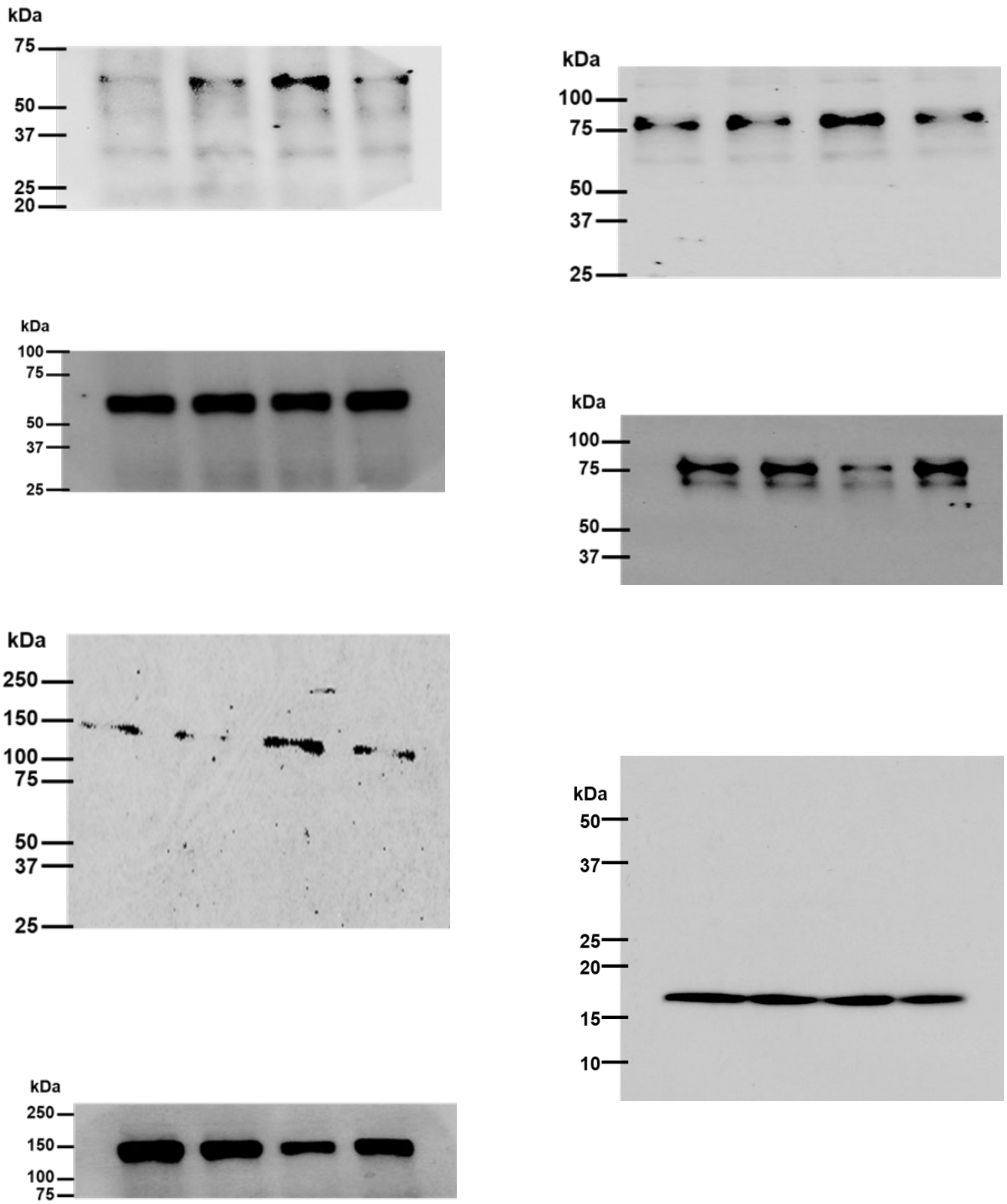
kDa
75
50
37
25
20
kDa
100
75
50
37
25
kDa
100
75
50
37
25
kDa
100
75
50
37
kDa
250
150
100
75
50
37
25
kDa
50
37
25
20
15
10
kDa
250
150
100
75

Fig. 8D

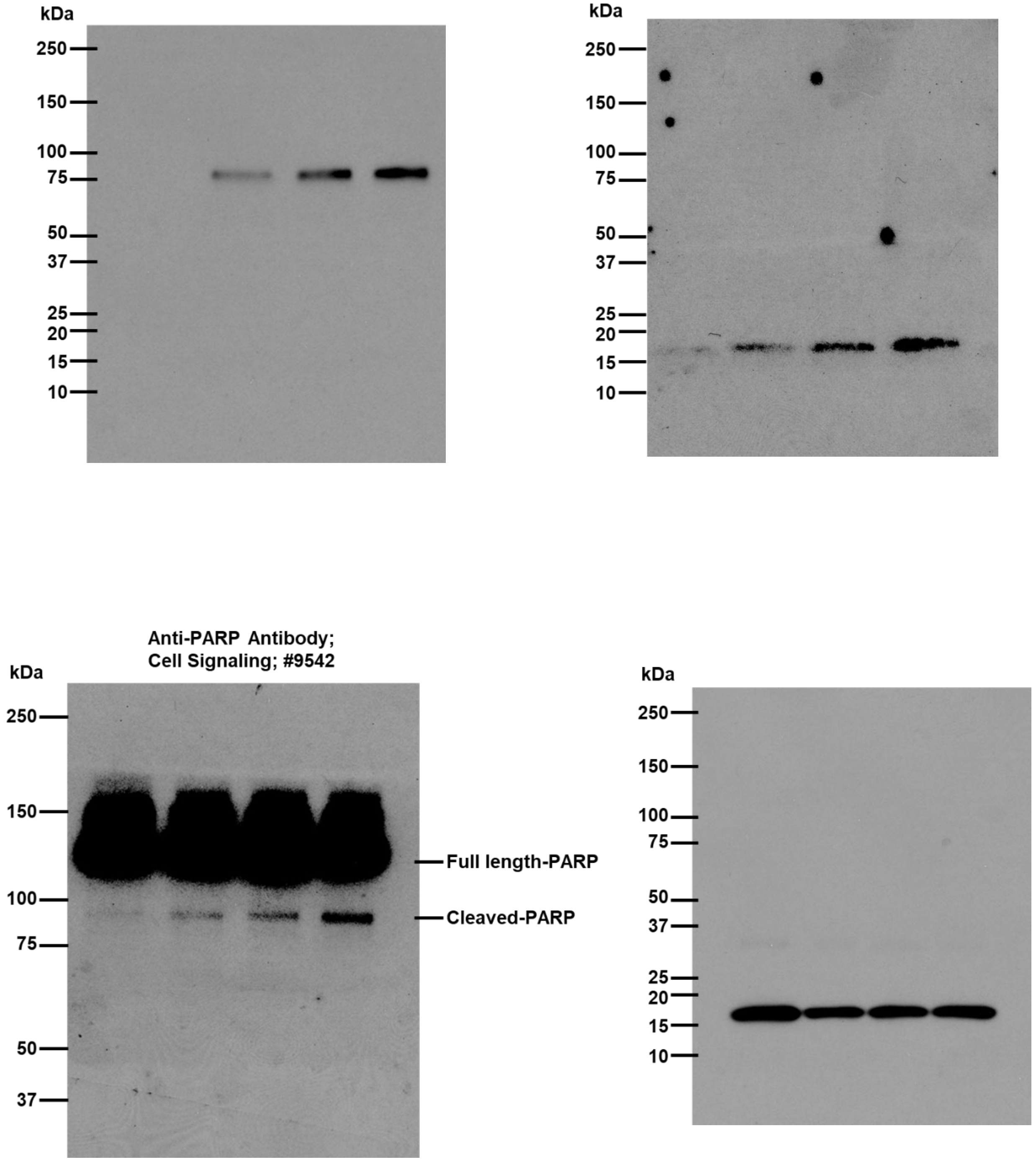
kDa
250
150
100
75
50
37
25
20
15
10
kDa
250
150
100
75
50
37
25
20
15
10
Anti-PARP Antibody;
Cell Signaling; #9542
kDa
250
150
100
75
50
37
Full length-PARP
Cleaved-PARP
kDa
250
150
100
75
50
37
25
20
15
10

Fig. 8F

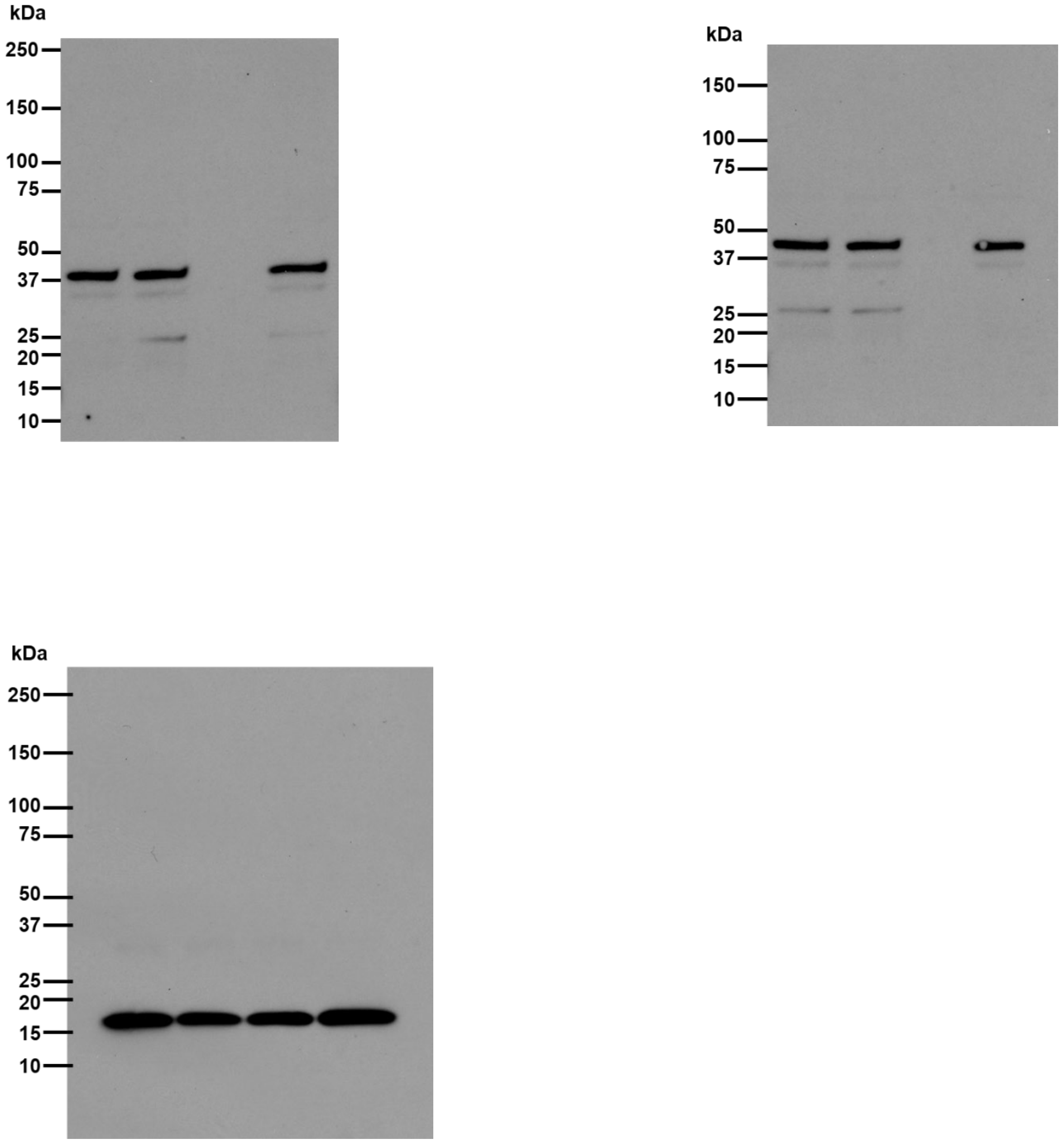

kDa
250
150
100
75
50
37
25
20
15
10
kDa
150
100
75
50
37
25
20
15
10
kDa
250
150
100
75
50
37
25
20
15
10
